# Significantly Enhanced Molecular Stacking in Ternary Bulk Heterojunctions Enabled by an Appropriate Side Group on Donor Polymer

**DOI:** 10.1002/advs.201903455

**Published:** 2020-02-16

**Authors:** Huanxiang Jiang, Xiaoming Li, Huan Wang, Zhitao Ren, Nan Zheng, Xunchang Wang, Yonghai Li, Weichao Chen, Renqiang Yang

**Affiliations:** ^1^ Key Laboratory of Optoelectronic Chemical Materials and Devices (Ministry of Education) School of Chemical and Environmental Engineering Jianghan University Wuhan 430056 China; ^2^ CAS Key Laboratory of Bio‐Based Materials Qingdao Institute of Bioenergy and Bioprocess Technology Chinese Academy of Sciences Qingdao 266101 China; ^3^ Center of Materials Science and Optoelectronics Engineering University of Chinese Academy of Sciences Beijing 100049 China; ^4^ College of Textiles & Clothing State Key Laboratory of Bio‐Fibers and Eco‐Textiles Collaborative Innovation Center for Eco‐Textiles of Shandong Province Qingdao University Qingdao 266071 China; ^5^ Zhengzhou Vocational College of Finance and Taxation Zhengzhou 450000 China; ^6^ Institute of Polymer Optoelectronic Materials and Devices State Key Laboratory of Luminescent Materials and Devices South China University of Technology Guangzhou 510640 China

**Keywords:** complementary absorption, molecular stacking, polymer solar cells, side chain effect, ternary bulk heterojunctions, ternary systems

## Abstract

Ternary strategy is a promising approach to broaden the photoresponse of polymer solar cells (PSCs) by adopting combinatory photoactive blends. However, it could lead to a more complicated situation in manipulating the bulk morphology. Achieving an ideal morphology that enhances the charge transport and light absorption simultaneously is an essential avenue to promote the device performance. Herein, two polymers with different lengths of side groups (P1 is based on phenyl side group and P2 is based on biphenyl side group) are adopted in the dual‐acceptor ternary systems to evaluate the relationship between conjugated side group and crystalline behavior in the ternary system. The P1 ternary system delivers a greatly improved power conversion efficiency (PCE) of 13.06%, which could be attributed to the intense and broad photoresponse and improved charge transport originating from the improved crystallinity. Inversely, the P2 ternary device only exhibits a poor PCE of 8.97%, where the decreased device performance could mainly be ascribed to the disturbed molecular stacking of the components originating from the overlong conjugated side group. The results demonstrate a conjugated side group could greatly determine the device performance by tuning the crystallinity of components in ternary systems.

## Graphical abstract

Side group effect in ternary polymer solar cells is studied by adopting polymers with different side groups. With appropriate side group modification, high power conversion efficiency (PCE) over 13% is realized, which could mainly be attributed to the broadened photoresponse and optimized molecular stacking. The results demonstrate that side group plays a crucial role in determining the molecular stacking of ternary heterojunction.

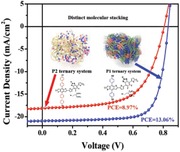

## 1 Introduction

Harvesting solar energy has been attracting extensive attention arousing from the environmental problem and the reserves of fossil fuels. Polymer solar cells (PSCs) have been emerged as one of the promising technologies for utilizing solar energy due to their advantages of flexibility, light weight, and possible semitransparence.^[^[qv: 1–11]^]^ Typical bulk heterojunction (BHJ) PSCs, composed of one donor and one acceptor, have demonstrated power conversion efficiency (PCE) over 15% with the rapid development of high‐performance donor and nonfullerene acceptor materials.^[^[qv: 12–17]^]^

Although immense progress has been made, the inherent narrow absorption spectra of organic semiconductors makes it hard to harvest a large portion of incident photons in the solar spectrum, which hinders the photocurrent and the improvement of device performance. Therefore, ternary PSCs composed of multiple donors or acceptors have emerged as a promising strategy to address this problem. The third component usually possesses complementary absorption with the host binary system and appropriate energy level alignment for efficient exciton dissociation.^[^[qv: 18–22]^]^ More importantly, the compatibility of components should be reasonably good to form an ideal bulk morphology that are conducive for charge transport and collection.^[^[qv: 23–27]^]^ With the prosperity of ITIC and its derivatives, abundant nonfullerene acceptors have been designed, which greatly increased the scope of material selection.^[^[qv: 28–35]^]^ Moreover, the structural similarity of ladder type acceptors usually exhibit good compatibility. Consequently, series of high performance ternary PSCs based on two nonfullerene acceptors have been reported.^[^[qv: 36–41]^]^ Yan and co‐workers fabricated ternary PSCs with PCE over 14% by using ITCPTC and MelC as acceptors.^[^[qv: 42]^]^ Zhang and co‐workers demonstrated a high performance ternary PSC with PCE of 16% by combining IT‐4F and Y6 as acceptors.^[^[qv: 43]^]^

So far, 2D BDT (2D‐BDT) have been widely adopted in donor materials because their intense intermolecular π–π interaction, strong absorption in visible region, and good hole mobility.^[^[qv: 44–47]^]^ We have reported a promising polymer donor with phenyl side group modified BDT (named PBTA‐PS), which delivered high PCE of 11.83% with ITIC as acceptor.^[^[qv: 48]^]^ After introducing 20% IT‐4F (weight ratio) into the PBTA‐PS:ITIC system, the ternary PSC shows simultaneously increased *J*_SC_ and FF, resulting in a high PCE of 13.27%. The improvement of device performance could be attributed to the broadened photoresponse, optimized molecular packing and the good compatibility of three components.^[^[qv: 49]^]^ To further delocalize the π electron cloud, enhance the intermolecular π–π interaction and charge transport, a polymer with biphenyl side group modified BDT (named PBDTTz‐SBP) was designed and synthesized by our group. When blended with ITIC, the PSC delivered a PCE of 12.09%, which is higher than the PBTA‐PS:ITIC system, indicating that attaching biphenyl side group to BDT is an effective approach to enhance intermolecular π–π interaction in binary system.^[^[qv: 50]^]^ However, whether it would take effect in ternary system is still unknown since the molecular packing and interaction of components are more complicated. This persuade us to systematically study the impact of different conjugated side group on the compatibility and crystallinity in ternary system to gain an insight into the relationship between chemical structure and ternary bulk morphology.

In this contribution, polymer PBTA‐PS (labeled as P1) with phenyl side group and PBDTTz‐SBP (labeled as P2) with biphenyl side group were selected as donors to study the side group effect in ternary system. IDIC‐C4Ph (labeled as LA1), a nonfullerene acceptor that shows the potential to balance the miscibility and crystallinity with donors, was selected as one acceptor in this work. The LA1 exhibits complementary absorption with P1 and P2 and the onset of absorption locates at 765 nm, which leaves sufficient room for broadening the photon harvesting.^[^[qv: 51]^]^ As to form the complementary absorption, NCBDT‐4Cl, a narrow bandgap acceptor with intense absorption in near‐infrared (NIR) region and appropriate energy level alignment was selected as another acceptor to construct ternary PSCs.^[^[qv: 52]^]^ Impressively, the P1 ternary PSC delivered a high PCE of 13.06%, which is 30% higher than the P1 binary devices. The greatly improved device performance could be mainly attributed to the broadened photoresponse, good compatibility of components, enhanced crystallinity and optimized charge transport. On the contrary, the P2 ternary PSC only exhibits a low PCE of 8.97%, which could be mainly ascribed to the less ordered molecular stacking of the components and the poor charge transport. Our results demonstrated that although introducing biphenyl side group could be an effective approach to enhance the intermolecular π–π interaction in binary system, it is a different circumstance in ternary system. The overlong conjugated side group on donor backbone could strongly affect the crystallinity and the resultant charge transport property in ternary system. Therefore, selecting donor polymer with appropriate length of side group could be of vital importance in realizing high performance ternary PSCs.

## 2 Results and Discussions

**Figure 1:**
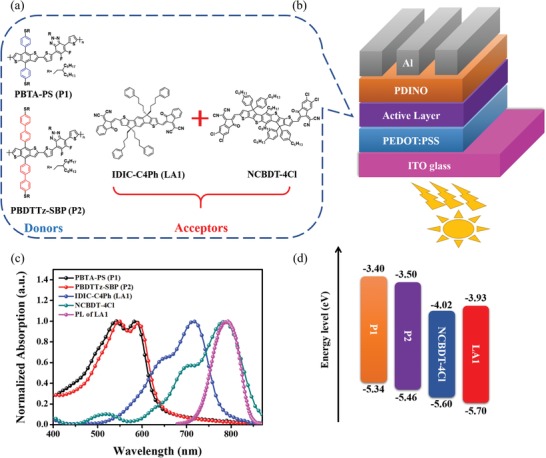
a) The chemical structure of donors and acceptors in this work. b) The device structure of binary and ternary PSCs. c) Normalized absorption spectra of neat P1, P2, LA1, and NCBDT‐4Cl films and photoluminescence (PL) spectrum of LA1. d) The energy level diagram of the components.

The chemical structures of the donors and acceptors in this work are shown in **Figure** **1**a. The corresponding absorption and energy levels are displayed in Figure 1c,d. P1 and P2 exhibit similar absorption region, which could form complementary absorption with LA1 and NCBDT‐4Cl. Therefore, the ternary systems could harvest a broad range of photons from 450 to 850 nm, which covers a wide range of solar spectrum. The highest occupied molecular orbitals (HOMO) energy levels of P1, P2, LA1, and NCBDT‐4Cl locate at −5.34, −5.46, −5.60, and −5.70 eV and the lowest unoccupied molecular orbitals (LUMO) lie on −3.40, −3.50, −4.02, and −3.93 eV, respectively. As a consequence, the driving force for exciton dissociation between donor and acceptors should be enough.^[^[qv: 49–52]^]^

**Figure 2:**
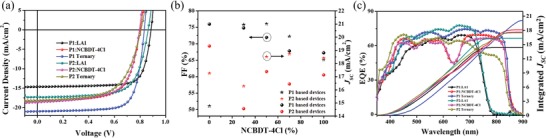
a) The current density–voltage (*J*–*V*) of optimal binary and ternary PSCs. b) The FF and *J*_SC_ of ternary PSCs at different weight ratios of NCBDT‐4Cl. c) EQE and integrated *J*_SC_ of binary and ternary devices.

Series of binary and ternary devices were fabricated to investigate the photovoltaic performance by adopting the conventional device structure of indium tin oxide (ITO)/poly(3,4‐ethylenedioxythiophene):poly(styrene sulfonate) (PEDOT:PSS)/Donor:Acceptors/perylene diimide functionalized with amino N‐oxide (PDINO)/aluminum (Al) (see Figure 1b). The optimal current density–voltage (*J*–*V*) curves and corresponding photovoltaic parameters are shown in **Figure** **2** and **Table** **1**. The P1:LA1 binary device delivered a PCE of 9.95% with a high *V*_OC_ of 0.88 V, a *J*_SC_ of 14.76 mA cm^−2^, and an excellent FF of 75.98%. The P2:LA1 device exhibits a higher PCE of 10.30% with an improved *J*_SC_ of 17.25 mA cm^−2^ and a moderate FF of 69.31%. The P1:NCBDT‐4Cl device shows a moderate PCE of 9.83% and the P2:NCBDT‐4Cl device exhibits a comparable PCE of 9.35%. With the introducing NCBDT‐4Cl into P1:LA1 blends, the FF gradually decreased and the *J*_SC_ increased first and then decreased in the ternary device. When NCBDT‐4Cl account for 30% of the acceptors (weight ratio, hereinafter), device performance of ternary device reached optimum with a high PCE of 13.06%, a greatly improved *J*_SC_ of 20.95 mA cm^−2^ and an outstanding FF approaching 75% (see Table S1 in the Supporting Information), which is much higher than the P1 based binary devices. On the contrary, after introducing 50% of NCBDT‐4Cl into the acceptors, the P2 ternary device delivered an optimal PCE of 8.97% with a low *J*_SC_ of 18.21 mA cm^−2^ and an inferior FF of 61.56% (see Table S2 in the Supporting Information), which is inferior to the P2:NCBDT and P2:LA1 binary devices. It should be noticed that the *V*_OC_'s of ternary devices are quite different with their binary counterparts. On the one hand, it is plausible to correlate the *V*_OC_ with the morphology of active layer since the donor–acceptor distance and crystallinity could affect *V*_OC_. On the other hand, the variation of *V*_OC_ in ternary devices also could be explained by the different operating models. In P1 ternary system, the *V*_OC_ gradually decreases with increasing the content of NCBDT‐4Cl, which could be explained by the alloy structure model. On the contrary, the *V*_OC_ of P2 ternary system hardly changes with the content of NCBDT‐4Cl, which could be correlated with the cascade structure model (see Tables S1 and S2 in the Supporting Information).^[^[qv: 21,53–56]^]^ Obviously, the P1 and P2 binary PSCs exhibit comparable device performance and P2:LA1 even shows a higher PCE than P1:LA1, which indicates that biphenyl side group could be a good candidate for constructing high performance binary PSC than phenyl side group. However, distinct device performance of P1 and P2 based ternary PSCs were observed, which could be attributed to the disturbed micromorphology induced by the extended entanglement of polymer chains.

**Table 1:** Device parameters of optimized binary and ternary PSCs

Active layers^a)^	*V*_OC_ [V]	*J*_SC_	*J*_SC_^EQE^	FF [%]	PCE [%]	μ_e_/μ_h_ [10^−4^ cm V^−1^ s^−1^]
		[mA cm^−2^]				
P1:LA1	0.88 (0.88 ± 0.01)	14.76 (14.45 ± 0.39)	14.37	75.98 (75.02 ± 1.02)	9.95 (9.61 ± 0.38)	1.68/1.48
P1:NCBDT‐4Cl	0.80 (0.80 ± 0.01)	18.29 (17.73 ± 0.54)	18.00	67.24 (66.83 ± 0.75)	9.83 (9.39 ± 0.55)	1.33/0.95
P1 Ternary	0.83 (0.83 ± 0.01)	20.95 (19.89 ± 0.89)	20.08	74.80 (74.54 ± 0.48)	13.06 (12.84 ± 0.30)	3.79/3.26
P2:LA1	0.86 (0.86 ± 0.01)	17.25 (16.56 ± 0.74)	16.74	69.31 (68.98 ± 0.46)	10.30 (9.93 ± 0.41)	1.40/1.06
P2:NCBDT‐4Cl	0.78 (0.78 ± 0.01)	18.40 (17.85 ± 0.73)	17.58	65.20 (65.01 ± 0.22)	9.35 (9.18 ± 0.39)	1.54/0.93
P2 Ternary	0.80 (0.80 ± 0.01)	18.21 (17.92 ± 0.28)	17.30	61.56 (59.95 ± 0.62)	8.97 (8.77 ± 0.36)	0.02/0.09

a) Average values with standard deviations were obtained from 20 cells.

The external quantum efficiency (EQE) measurement was conducted to gain an insight into the photoresponse of binary and ternary PSCs at different wavelengths. As shown in Figure 2c, the P1:LA1 and P2:LA1 devices exhibit strong and uniform photoresponse between 400 and 750 nm with maximum EQE over 65%. The P1:NCBDT‐4Cl and P2:NCBDT‐4Cl devices show broad photoresponse between 400–850 nm. However, a deep trough can be observed between 600–700 nm, which could be attributed to the absence of absorption. Therefore, NCBDT‐4Cl could be a promising third component adding into P1/P2:LA1 system to broaden the scope of solar harvesting. As a result, the P1 ternary device demonstrated high and flat photoresponse between 400–850 nm with maximum EQE over 70% and the deep trough in the P1:NCBDT‐4Cl system is effectively paved over. Inversely, though broad photoresponse was realized in P2 ternary device, the photoresponse was weakened in the whole spectrum, which could be attributed to the sabotaged molecular packing and the undesirable charge transport in P2 based ternary blend. The *J*_SC_'s calculated from EQE curves are consistent with the measured values with little error (<5%) (see Figure 2c and Table 1).

The interactions between acceptors are of vital importance to understand the working mechanism of ternary devices. First, acceptor‐only devices were fabricated with the device structure of ITO/PEDOT:PSS/Acceptors/PDINO/Al to evaluate the possibility of charge transfer between the acceptors. As shown in Figure S2 in the Supporting Information, the *J*_SC_ of LA1, NCBDT‐4Cl, and LA1:NCBDT‐4Cl devices are 0.37, 0.12, and 0.09 mA cm^−2^, respectively, indicating no significant charge transport between acceptors. Subsequently, photoluminescence (PL) of neat LA1, NCBDT‐4Cl and their blend films were conducted to see if there is energy transfer between LA1 and NCBDT‐4Cl since the PL spectrum of LA1 perfectly overlaps with the absorption of NCBDT‐4Cl, which could be a spectral indication of energy transfer from LA1 to NCBDT‐4Cl (see Figure 1c). The neat LA1 film exhibits strong PL emission (excited at 680 nm) and the NCBDT‐4Cl film shows relatively weak PL emission (excited at 770 nm). After blending LA1 with NCBDT‐4Cl, the blend film only shows one characteristic emission peak around 827 nm, which is the same as the PL peak of NCBDT‐4Cl (excited at 680 nm). Moreover, the emission peak of LA1 completely vanished, indicating that there is efficient energy transfer from LA1 to NCBDT‐4Cl. Consequently, LA1 excitons could be transferred to NCBDT‐4Cl by the energy transfer and then dissociated to free charge carrier.

**Figure 3:**
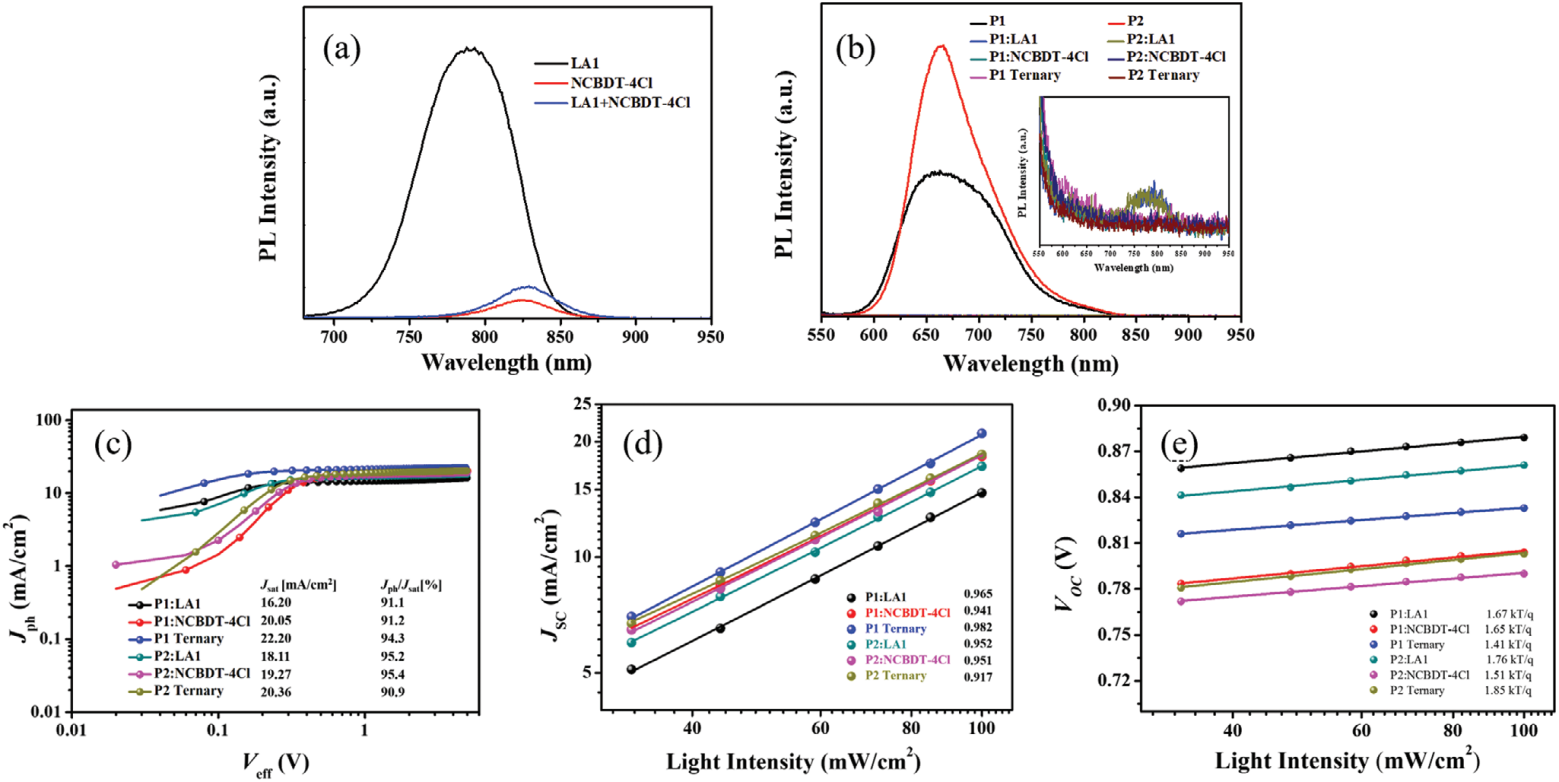
a) PL spectra of neat LA1, NCBDT‐4Cl, and their blend films. b) PL spectra of P1 and P2 based blend films. (The inset graph represents the amplification of the tiny signals.) c) *J*_ph_ versus *V*_eff_. d) *J*_SC_'s measured under different light intensity. e) *V*_OC_ versus light intensity.

PL quenching was further conducted to study the exciton dissociation process. As shown in **Figure** **3**b, neat P1 and P2 films exhibit strong PL emission, after blended with acceptors, most of the PL are effectively quenched in the binary and ternary blend films, indicative of sufficient contact surface for exciton dissociation (excited at 550 nm). Our result demonstrated that the exciton dissociation was not the main reason for the drastic different performance of P1 and P2 ternary devices.^[^[qv: 24,57–58]^]^ Consequently, the relationship between photocurrent density (*J*_ph_) and effective voltage (*V*_eff_) were plotted to gain a further insight into charge transport and collection in optimized binary and ternary devices. *J*_ph_ = *J*_L_ − *J*_D_, where *J*_L_ and *J*_D_ refer to the current density measured under AM 1.5G illumination and in dark, respectively. The *V*_eff_ is calculated by *V*_0_ − *V*, where *V*_0_ refers to the voltage that *J*_ph_ = 0 and *V* represents the applied voltage. As shown in Figure 3c, the *J*_ph_'s of both P1 and P2 based devices reached saturation values (*J*_sat_) at a low *V*_eff_ < 1 V, indicating most of the generated charges were driven to the electrodes. It should be noticed that the *J*_ph_ of P1 ternary device reached saturation at smaller *V*_eff_ and shows larger *J*_sat_ than P2 ternary device, indicating more charge carriers were collected at the electrodes in P1 ternary system and the charge transport could be more efficient. What is more, the *J*_ph_/*J*_sat_ ratio under short circuit condition could be used to evaluate the overall charge collection efficiency. (see Figure 3c). The P2 binary devices exhibits higher value than P1 binary devices, indicating biphenyl side group could help promoting charge transport in binary system due to the intense intermolecular π–π interaction. Inversely, the P1 ternary devices shows higher value than P2 ternary devices, demonstrating improved charge transport, which could be a sign of more favorable transport pathway. The bimolecular recombination behavior of binary and ternary devices was studied by recording *J*_SC_ at different light intensities (*P*_light_). The relation of *J*_SC_ and *P*_light_ follows the power‐law dependence (*J*_SC_ ∝ *P*_light_ *^α^*), where α approaching 1 indicates negligible bimolecular recombination. As shown in Figure 3d, the bimolecular recombination was effectively suppressed in P1 ternary device. On the contrary, the P2 ternary device exhibit more severe bimolecular recombination than P2 binary devices. The *V*_OC_ versus light intensity mainly reflects the monomolecular recombination process. The slope approaching *kT*/*q* reflects less monomolecular recombination. Therefore, the *V*_OC_ versus light intensity of binary and ternary devices has been plotted. The slope of P1 ternary device is 1.41 *kT*/*q*, which is smaller than the binary counterparts, indicating the monomolecular recombination is effectively suppressed. On the contrary, P2 ternary device exhibits a large slope of 1.85 *kT*/*q*, demonstrating more serious monomolecular recombination (see Figure 2e).^[^[qv: 59]^]^ In conclusion, both P1 and P2 ternary device exhibit efficient exciton dissociation, but P1 ternary device shows more efficient charge transport, which could partly explain the high *J*_SC_'s and FFs in P1 ternary devices.

The mobilities of charge carriers were characterized by space charge‐limited current (SCLC) method. The electron‐only and hole‐only diodes were fabricated to evaluate the electron and hole mobilities by adopting the device structure of ITO/zinc oxide (ZnO)/Active layer/PDINO/Al and ITO/PEDOT:PSS/Active layer/Au, respectively. The P1 ternary device exhibits improved and more balanced charge transport compared with P1 binary devices, which are consistent with the high FF. Inversely, the P2 ternary device shows low and unbalanced charge carrier mobilities, indicative of nonideal charge transport (Table 1; Figure S3, Supporting Information), which might account for the low FF and *J*_SC_.

**Figure 4:**
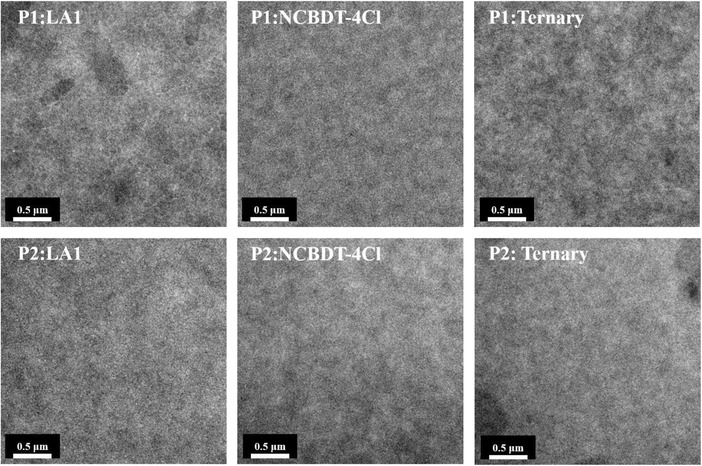
The TEM images of P1 and P2 based binary and ternary blend films.

To further explore the origin of exciton dissociation and charge transport process, it is essential to probe the micromorphology of the blend film. First, the bulk and surface morphology of P1 and P2 binary and ternary blend films were probed by transmission electron microscopy (TEM) and atomic force microscopy (AFM). The bright and dark regions could reflect the density distribution of the components. As shown in **Figure** **4** and Figure S4 (Supporting Information), P1 and P2 binary blend films exhibit homogeneous bulk morphology and smooth surface with low root mean square (RMS) around 1. After introducing NCBDT‐4Cl into the P1/P2:LA1 host system, the P1 and P2 ternary blend film still shows uniform distribution of components with no large aggregates observed, demonstrating the introduction of NCBDT‐4Cl did not disrupt the miscibility of the components. The components are well blended in ternary systems, enabling sufficient contact area between donor and acceptors, which corresponds well with the efficient exciton dissociation probed by PL.

Water contact angles (WCAs) measurement was performed to evaluate the surface energies (γ) of components since they can effectively reflect the compatibilities.^[^[qv: 38,60]^]^ The WCAs of pristine and blend films are shown in Figure S5 in the Supporting Information and the corresponding surface energies are summarized in Table S3 in the Supporting Information. WCAs of LA1 and NCBDT‐4Cl are 92° and 96°, respectively. After blending LA1 and NCBDT‐4Cl, the blend film exhibits larger WCA of 98° and a smaller surface energy of 24.31 mN m^−1^, indicative of the enlarged molecular distance and the weakened molecular interaction, which could be attributed to the good compatibility of acceptors. Moreover, the more similarity between the WCA of donor and acceptors could improve the compatibility of the components, resulting in efficient exciton dissociation, which is consistent with the TEM and AFM results.

**Figure 5:**
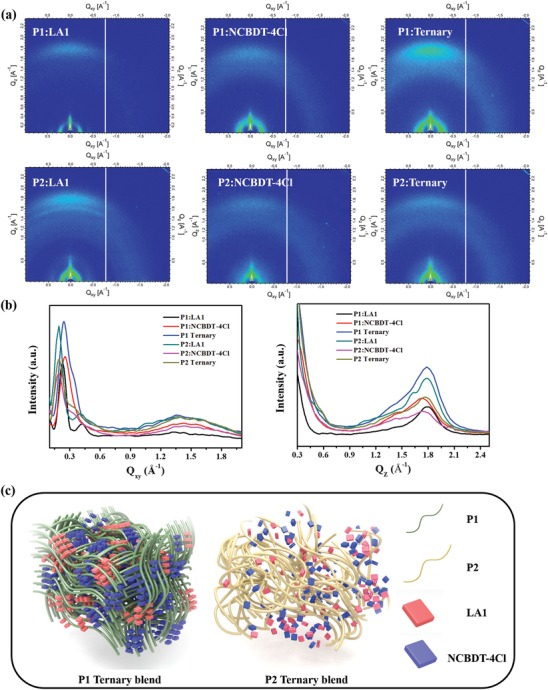
a) 2D‐GIXD patterns of P1 and P2 based binary and ternary films. b) The corresponding linecut profiles along the in plane and out of plane directions. c) The schematic diagram of crystalline behavior in ternary blends.

Furthermore, 2D grazing incidence X‐ray diffraction (2D‐GIXD) measurement was performed to study the orientation and crystallinity of components in thin films. The 2D‐GIXD patterns and corresponding line profiles along the in plane (IP) and out of plane (OOP) of neat and blend films are shown in Figure S6 in the Supporting Information and **Figure** **5**, respectively. The neat P1, P2, LA1, and NCBDT‐4Cl films show (100) diffraction peak at *q_xy_* = 0.23, 0.18, 0.40, and 0.30 Å^−1^ and (010) diffraction peak at *q_z_* = 1.76, 1.75, 1.84, and 1.82 Å^−1^, respectively, indicating the donors and acceptors adopt face‐on orientation in neat films. The P1:LA1 and P2:LA1 films exhibit characteristic (100) peaks of polymers and LA1 along the IP direction and a combined (010) diffraction peak along the OOP direction, indicating that P1, P2, and LA1 adopted the preferential face‐on orientation in the binary films. The P2:LA1 film exhibits much stronger crystallinity than P1:LA1, and P2:NCBDT‐4Cl shows comparable crystallinity with P1:NCBDT‐4Cl film. However, distinct crystalline behaviors were observed in ternary film after introducing NCBDT‐4Cl into the polymer:LA1 system. The P1 ternary film exhibits remarkably enhanced (100) and (010) diffraction peak along the IP and OOP direction, respectively, indicating that incorporating NCBDT‐4Cl into Polymer:LA1 system did not destroy the original face‐on orientation of the components and it could function as a crystalline modulator to optimize the molecular packing. On the contrary, the P2 ternary shows drastically decreased (010) peak along the OOP direction, indicating the less ordered molecular packing. Moreover, the π–π stacking distances of P1 and P2 ternary blend films are 3.52 and 3.57 Å, respectively. Besides, the crystalline coherence length (CCL) of π–π stacking along OOP direction in P1 and P2 ternary blend films are 13.84 and 10.05 Å, respectively. These results indicate that the molecular stacking in P1 ternary film is more ordered and compact than in P2 ternary film, which is beneficial for charge transport in vertical direction.

Therefore, combined with the TEM and AFM result, it can be inferred that biphenyl side group on BDT could did not induce oversize phase separation but could disrupt the ordered molecular stacking in the ternary system (Figure 5c). The molecular stacking behaviors in ternary devices could mainly account for the distinct charge transport properties of P1 and P2 ternary system.

## 3 Conclusions

In summary, series of binary and ternary PSCs were fabricated by adopting P1 and P2 as donors, LA1 and NCBDT‐4Cl as acceptors to gain an insight into the side group effect on crystallinity in ternary polymer solar cells. Although the P1 and P2 binary devices show comparable photovoltaic performance and P2:LA1 even exhibits a higher PCE than P1:LA1, the results are sharply different in the ternary systems. The P1 ternary devices shows an obviously improved PCE of 13.06% with a *V*_OC_ of 0.83 V, a high *J*_SC_ of 20.95 mA cm^−2^, and an outstanding FF of 74.80%. The improved device performance could be attributed to the broadened and intense photoresponse, good compatibility of components, efficient charge transport, and the more ordered molecular stacking. Unexpectedly, the P2 ternary device shows a low PCE of 8.97% with a *V*_OC_ of 0.80 V, a moderate *J*_SC_ of 18.21 mA cm^−2^ and an inferior FF of 61.56%. The undesirable device performance could be mainly attributed to the disrupted molecular stacking of the components induced by the extended conjugated side group on BDT. In conclusion, conjugated side group on donor backbone could clearly affect the crystalline behavior in ternary system and determine the device performance. Therefore, it is essential to choose polymers with appropriate conjugated side group to construct high performance ternary PSCs.

## Conflict of Interest

The authors declare no conflict of interest.

## Supporting information

Supporting Information
