# Supplementary material for: Significantly Enhanced Molecular Stacking in Ternary Bulk Heterojunctions Enabled by an Appropriate Side Group on Donor Polymer
Source: Adv Sci (Weinh). 2020 Feb 16;7(7):1903455. doi: 10.1002/advs.201903455 (PMC7141074; doi:10.1002/advs.201903455)
Supplement: Supplementary file 1 — Supporting Information [file ADVS-7-1903455-s001.pdf]

Copyright WILEY-VCH Verlag GmbH & Co. KGaA, 69469 Weinheim, Germany, 2020.

ADVANCED SCIENCE
Open Access

Supporting Information

for *Adv. Sci.,* DOI: 10.1002/advs.201903455

Significantly Enhanced Molecular Stacking in Ternary Bulk Heterojunctions Enabled by an Appropriate Side Group on Donor Polymer

Huanxiang Jiang, Xiaoming Li, Huan Wang, Zhitao Ren, Nan Zheng, Xunchang Wang, Yonghai Li,* Weichao Chen,* and Renqiang Yang**

Copyright WILEY-VCH Verlag GmbH & Co. KGaA, 69469 Weinheim, Germany, 2020.

Supporting Information

Significantly enhanced molecular stacking in ternary bulk heterojunction enabled by appropriate side group on donor polymer

Huanxiang Jiang, Xiaoming Li, Huan Wang, Zhitao Ren, Nan Zheng, Xunchang Wang, Yonghai Li*, Weichao Chen* and Renqiang Yang**

H. Jiang, Prof. R. Yang
Key Laboratory of Optoelectronic Chemical Materials and Devices (Ministry of Education), School of Chemical and Environmental Engineering, Jianghan University
Wuhan 430056, China
Email: yangrq@qibebt.ac.cn (R.Y.)

H. Jiang, X. Li, X. Wang, Dr. Y. Li, Prof. R. Yang
CAS Key Laboratory of Bio-based Materials, Qingdao Institute of Bioenergy and Bioprocess Technology, Chinese Academy of Sciences,
Qingdao 266101, China
Email: liyh@qibebt.ac.cn (Y. L.)

H. Wang, Dr. W. Chen
College of Textiles & Clothing, State Key Laboratory of Bio-fibers and Eco-textiles, Collaborative Innovation Center for Eco-Textiles of Shandong Province, Qingdao University
Qingdao 266071, China
Email: chenwc@qdu.edu.cn (W. C.)

Dr. N. Zheng
Institute of Polymer Optoelectronic Materials and Devices, State Key Laboratory of Luminescent Materials and Devices, South China University of Technology
Guangzhou 510640, China
Email: zhengn@scut.edu.cn (N. Z.)

Z. Ren
Zhengzhou Vocational College of Finance and Taxation
Zhengzhou 450000, China

H. Jiang
Center of Materials Science and Optoelectronics Engineering, University of Chinese Academy of Sciences
Beijing 100049, China

Instruments and measurements

UV-vis absorption spectra were performed on Lambda 25 spectrophotometer. Current density-voltage (*J-V*) curves of the solar cells were characterized by a Keithley 2420 source

meter. A standard silicon solar cell was used to calibrate the light intensity. The external quantum efficiencies (EQEs) of devices were measured using a certified Newport incident photon conversion efficiency (IPCE) measurement system. Atomic force microscopy (AFM) was performed by tapping mode under an argon atmosphere, using an Agilent 5400 instrument. Transmission electron microscopy (TEM) were performed by a HITACHI H-7650 electron microscope at an accelerate voltage of 100 kV. DSC measurement was performed by DSC Q2000 V24.11 Build 124 at a heating rate of 10°C/min under a nitrogen atmosphere. Two dimensional grazing incidence X-ray diffraction (2D-GIXD) were performed on Xeuss 2.0. The X-ray source was MetalJet-D2, Excillum and detector was Pilatus3R 1M, Dectris. The water contact angle (WCA) measurement was performed on JY-PHb.

Material and reagents

P1, P2, LA1 were synthesis by our group according to the reference.[1,2] NCBDT-4Cl was purchased from Solarmer Materials Inc. Other chemicals were all purchased commercially and used as received.

Fabrication and characterization of devices

The binary and ternary polymer solar cells (PSCs) were fabricated using the conventional device structure of indium tin oxides (ITO) glass/ poly(3,4-ethylenedioxythiopene):poly(styrenesulfonate) (PEDOT:PSS)/Donor:Acceptors/perylene diimide functionalized with amino N-oxide (PDINO)/Al. The ITO glasses were sequentially cleaned in detergent, demonized water, acetone and isopropanol ultrasonically for 13 min each step. Then the ITO glasses were treated with oxygen plasma for 6 min. The plasma treated ITO glasses were then spin coated with PEDOT:PSS (Baytron PVP Al 4083) and followed by annealing at 160°C for 20 min. After that, the ITO glasses were transferred to a glove with nitrogen atmosphere. The chlorobenzene (CB) solutions (10 mg/mL for polymer) with different ratios of acceptors were stirred at room temperature for 5 hours before spin coating. Additive (DPE) was added into the ternary blend solution 1 hour before spin coating.

The binary and ternary blend films were spin coated on the ITO substrate as the active layers (~100 nm) at different speed. Afterwards, PDINO, the electron transporting layer was spin coated onto the active layer at 3000 rpm for 10 s. Finally, the samples were transferred to a vacuum chamber for the deposition of Al (30 nm) at high vacuum (10^{-5} Pa). The active area for each cell is 0.1 cm^2. All PSCs were measured under illumination of an AM 1.5G solar light simulator at 100 mW/cm^2.

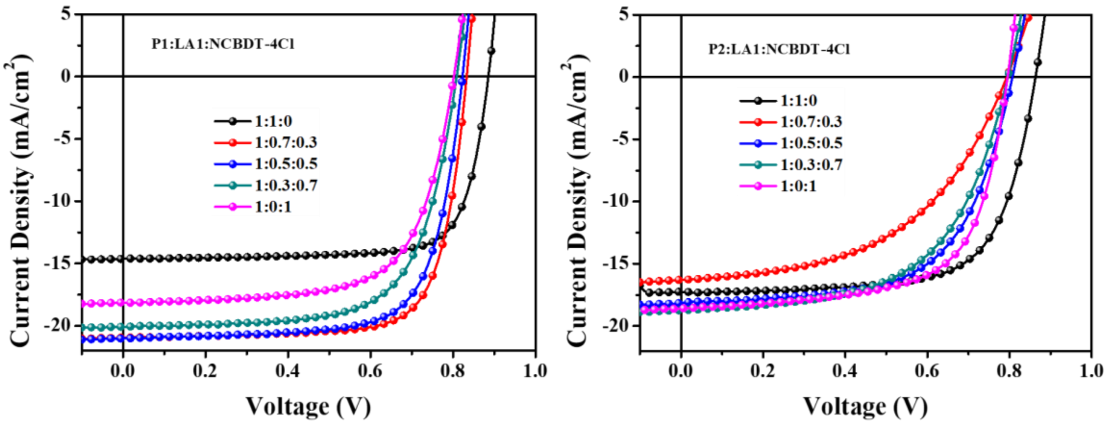

Figure S1. *J-V* curves of P1 and P2 ternary devices with various weight ratios.

Table S1. Device parameters of P1 based ternary PSC with different ratio of acceptors

P1:LA1:NCBDT-4Cl	V_{OC} [V]	J_{SC} [mA/cm^2]	FF [%]	PCE [%]
1:1:0	0.88 (0.88±0.01)	14.76 (14.45±0.39)	75.98 (75.02±1.02)	9.95 (9.61±0.38)
1:0.7:0.3	0.83 (0.83±0.01)	20.95 (19.89±0.89)	74.80 (74.54±0.48)	13.06 (12.84±0.30)
1:0.5:0.5	0.82 (0.82±0.01)	21.01 (20.58±0.59)	71.94 (71.67±0.32)	12.50 (12.11±0.40)
1:0.3:0.7	0.81 (0.81±0.01)	20.06 (19.66±0.44)	67.81 (67.48±0.41)	11.03 (10.59±0.68)
1:0:1	0.80 (0.80±0.01)	18.29 (17.73±0.54)	67.24 (66.83±0.75)	9.83 (9.39±0.55)

[a] Average values with standard deviations were obtained from 20 cells

Table S2. Device parameters of P2 based ternary PSC with different ratio of acceptors

P2:LA1:NCBDT-4Cl	V_{OC} [V]	J_{SC} [mA/cm^2]	FF [%]	PCE [%]
1:1:0	0.86 (0.86±0.01)	17.25 (16.56±0.74)	69.31 (68.98±0.46)	10.30 (9.93±0.41)
1:0.7:0.3	0.79 (0.79±0.01)	16.27 (15.93±0.32)	50.28 (74.54±0.48)	6.48 (6.32±0.28)
1:0.5:0.5	0.80 (0.80±0.01)	18.21 (17.92±0.28)	61.56 (59.95±0.62)	8.97 (8.77±0.36)
1:0.3:0.7	0.79 (0.79±0.01)	18.72 (18.36±0.46)	57.78 (57.48±0.31)	8.60 (8.31±0.45)
1:0:1	0.78 (0.78±0.01)	18.40 (17.85±0.73)	65.20 (65.01±0.22)	9.35 (9.18±0.39)

[a] Average values with standard deviations were obtained from 20 cells

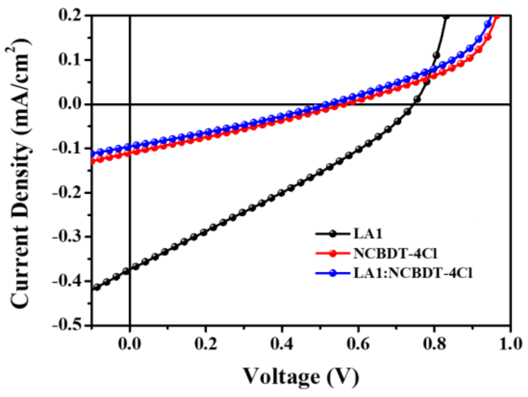

Figure S2. *J*-*V* curve of acceptor-only devices.

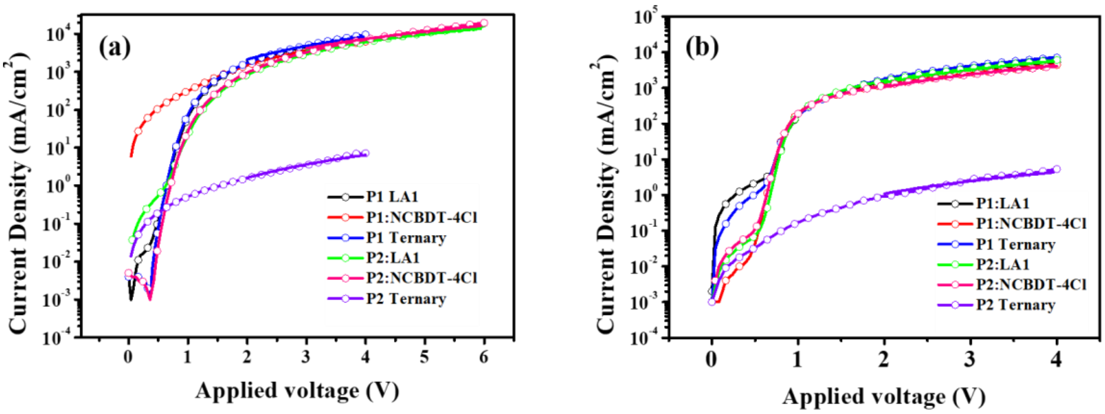

Figure S3. *J*-*V* curves of (a) electron-only and (b) hole-only diodes of binary and ternary devices.

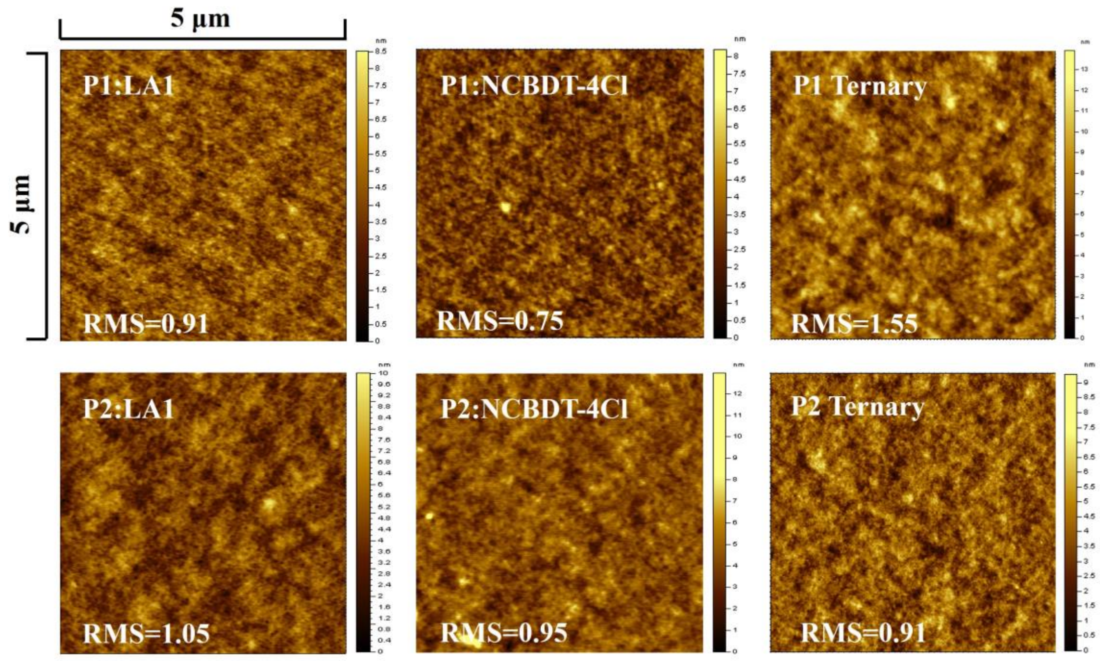

Figure S4. The AFM images of binary and ternary devices.

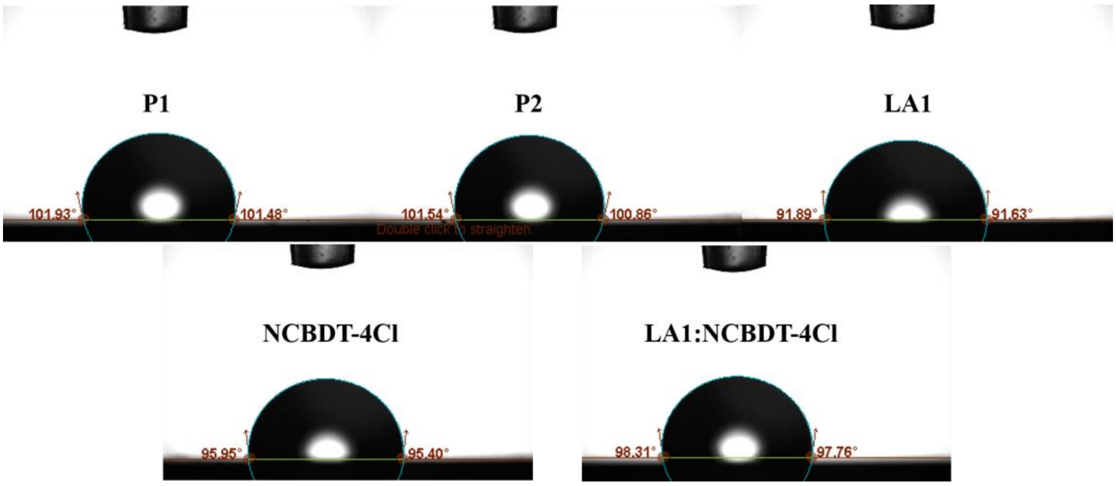

Figure S5. Water contact angles (WCAs) of neat donors, neat acceptors, and their blend films.

Table S3. Contact angle and surface energy of neat films and blend films

**Films**	**Water contact angle [°]**	**Surface energy γ [mN/m]**
P1	102	21.99
P2	101	22.32
LA1	92	28.12
NCBDT-4Cl	96	25.72
LA1:NCBDT-4Cl	98	24.31

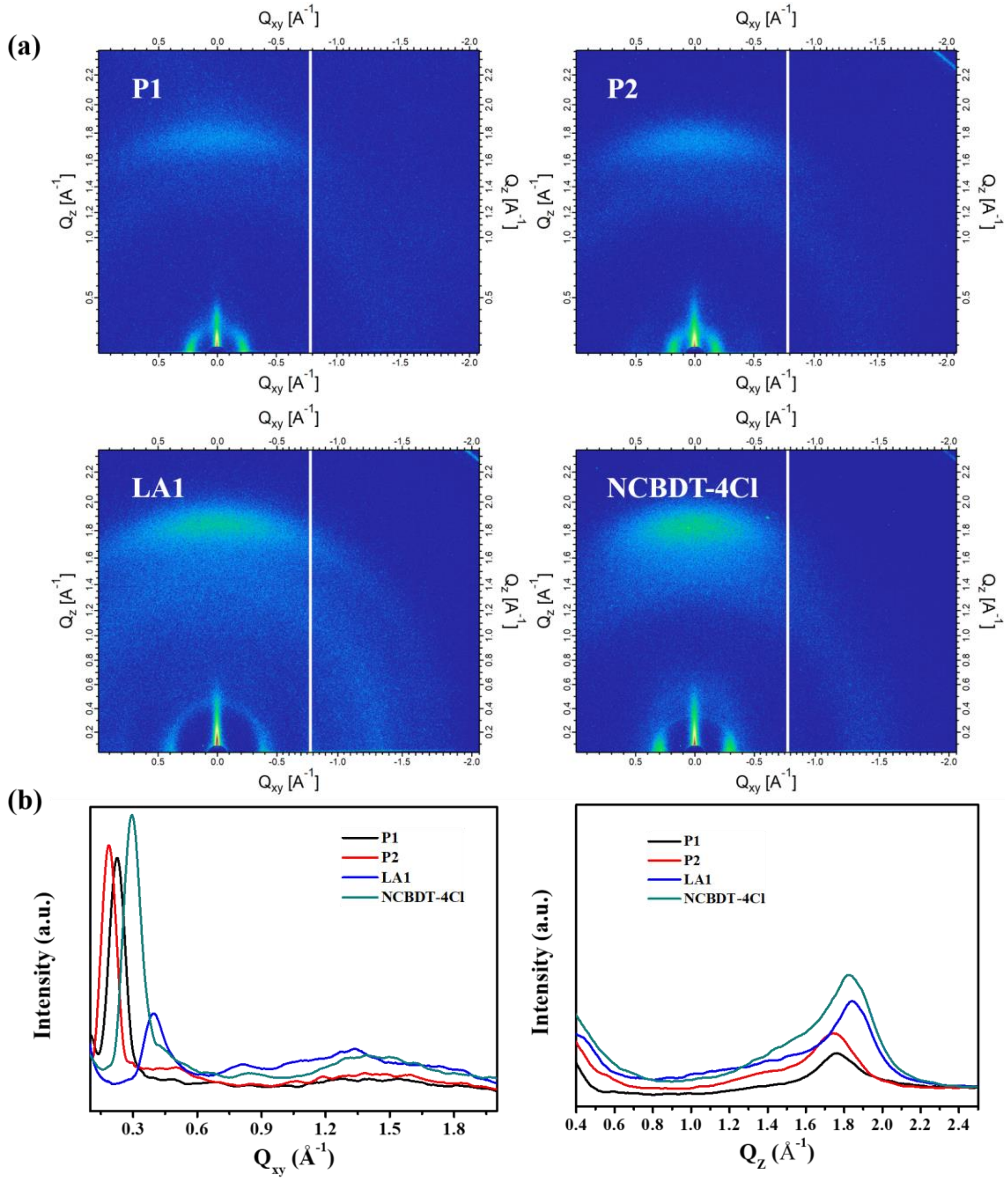

Figure S6. (a) 2D-GIXD patterns of neat donors and acceptors films.(b) The corresponding line cut profiles along the in plane and out of plane directions.

Reference:

1. X. Li, G. Huang, N. Zheng, Y. Li, X. Kang, S. Qiao, H. Jiang, W. Chen, R. Yang, *Sol. RRL*, **2019**, *3*, 1900005.
2. W. Chen, G. Huang, X. Li, H. Wang, Y. Li, H. Jiang, N. Zheng, R. Yang, *ACS Appl. Mater. Inter.*, **2018**, *10*, 42747.
